# Albumin–Bilirubin (ALBI) Score Predicts Long-Term Survival in Elderly Patients with Decompensated Heart Failure

**DOI:** 10.3390/jcm14030808

**Published:** 2025-01-26

**Authors:** Michał Jurkiewicz, Wioletta Szczurek-Wasilewicz, Michał Skrzypek, Sebastian Krych, Mariusz Gąsior, Bożena Szyguła-Jurkiewicz

**Affiliations:** 1 Student’s Scientific Society, 3rd Department of Cardiology, Faculty of Medical Sciences in Zabrze, Medical University of Silesia, 40-055 Katowice, Poland; 2 2nd Department of Cardiology and Angiology, Silesian Center for Heart Diseases, 41-800 Zabrze, Poland; 3 Department of Pharmacology, Faculty of Medicine, University of Opole, 45-040 Opole, Poland; 4 Department of Biostatistics, Faculty of Public Health in Bytom, Medical University of Silesia in Katowice, 40-055 Katowice, Poland; 5 Student’s Scientific Association, Department of Cardiac, Vascular and Endovascular Surgery and Transplantology, Faculty of Medical Sciences in Zabrze, Medical University of Silesia, 40-055 Katowice, Poland; 6 Department of Cardiac, Vascular and Endovascular Surgery and Transplantology, Faculty of Medical Sciences in Zabrze, Medical University of Silesia, 40-055 Katowice, Poland; 7 3rd Department of Cardiology, Faculty of Medical Sciences in Zabrze, Medical University of Silesia, 40-055 Katowice, Poland

**Keywords:** albumin–bilirubin ratio, risk stratification, heart failure, elderly patients

## Abstract

**Background/Objective**: Risk stratification in elderly patients with heart failure (HF) is very difficult. There is a lack of reliable tools for assessing the risk of death in this population of patients. The aim of this study was to determine the association between albumin–bilirubin (ALBI) score and long-term mortality in hospitalized elderly patients with decompensated HF. **Methods**: The study included 242 consecutive HF patients aged over 65 years hospitalized for worsening chronic HF at our institution between 2019 and 2023. The ALBI score was calculated according to the appropriate formula. **Results**: The median (IQR) age of the study population was 68.00 (range 66.0 to 74.6) years and 21.1% were female. The mean follow-up time was 352 ± 293 days. During the follow-up period, 47.1% patients died. The ALBI score generated good prognostic power (area under the curve = 0.822, *p* < 0.01) and specificity (86%) as well as acceptable sensitivity (68%) when predicting long-term mortality. Patients with higher ALBI scores (≥−2.191) had significantly worse long-term survival than patients with lower ALBI scores (<−2.191) [37 (25.2%) vs. 77 (81.1%); log rank *p* < 0.001]. **Conclusions**: This is the first study demonstrating that ALBI score has good prognostic power and allows for the successful prediction of mortality during long-term follow-up in the analyzed cohort.

## 1. Introduction

Despite modern therapeutic options, heart failure (HF) is an increasingly serious public health problem, particularly among older patients [1]. Although half of all patients with HF are over 75 years of age, most clinical trials evaluate much younger cohorts, and patients with advanced age are excluded from global analyses and are underrepresented in clinical registries. Therefore, the results of many studies cannot be unequivocally generalized to older patients and may require special considerations for older patients [2]. HF is closely related to liver function due to reduced cardiac output and increased central venous pressure [1,2,3]. An elevated serum bilirubin level is a marker of cholestasis and reflects liver congestion. In turn, a decreased serum albumin level is associated with impaired liver synthesizing function and consequently leads to malnutrition and cachexia [4]. Therefore, the combined albumin–bilirubin (ALBI) score may reflect the severity of cardio-hepatic syndrome and subsequent worse prognosis in HF [5,6]. The ALBI score was originally developed to assess the prognosis of patients with hepatocellular carcinoma, and since then it has become a valuable tool for assessing the prognosis of patients who have undergone radiofrequency ablation, heart valve surgery and radiotherapy, as well as in patients with acute kidney injury [7,8].

In the available literature, there are only a few studies that address the importance of ALBI in patients with HF [3,5,6,9]. Previous studies have shown that the ALBI score was a useful prognostic marker of mortality in patients with acute HF and a marker of response to cardiac resynchronization therapy in patients with HF. However, these studies usually analyzed other age groups of patients, and the percentage of patients with end-stage HF was relatively small, or included a relatively limited number of events due to the short follow-up period [3,5,6,9]. Although the ALBI score is considered to be a promising liver dysfunction indicator [5,9], its clinical and prognostic value has not been fully evaluated in elderly patients with advanced HF. Therefore, in this study, we aimed to determine the association between ALBI score and long-term mortality in elderly patients with end-stage HF hospitalized due to decompensated HF.

## 2. Material and Methods

This was a single-center observational study that included consecutive HF patients aged over 65 years hospitalized for worsening chronic HF at our institution between 2019 and 2023. All records of patients > 65 years of age who were admitted to our hospital for acute HF exacerbation were analyzed, and patients who met the inclusion criteria were included in the analysis. Exacerbation of HF was defined as HF requiring urgent therapy and hospitalization, based on the Framingham criteria. Inclusion criteria were as follows: age > 65 years, advanced HF optimally treated at least 3 months before inclusion in the study, New York Heart Association (NYHA) functional class III and IV. Exclusion criteria were as follows: acute coronary syndrome, acute myocarditis, infective endocarditis, presence of sepsis or malignancy, moderate to severe chronic obstructive pulmonary disease (COPD), severe kidney disease or severe liver disease (Child–Pugh score B or C). At the time of enrollment to the study, all patients underwent physical examination, anthropometric measurements, a panel of laboratory tests (hemoglobin, platelets, albumin, total bilirubin, aspartate aminotransferase, alanine aminotransferase, high-sensitivity C-reactive protein, creatinine, sodium and brain natriuretic peptide) and echocardiography.

The ALBI score was calculated as follows: (log10 total bilirubin [mmol/L] × 0.66) + (albumin [g/L] × −0.085) [8].

Patients’ death was confirmed based on the official registry of the National Health Fund or their medical record. The endpoint of the study was defined as death from any cause during long-term follow-up. Written informed consent was obtained from all study patients, and the study protocol was approved by the Medical University of Silesia Ethics Committee (specific ethics codes: KNW/0022/KB1/53/18, date of approval, 19 June 2018; and PCN/0022/KB1/20/I/21, date of approval, 4 January 2021).

### Statistical Analysis

Statistical tests were performed with SAS software version 9.4 (SAS Institute Inc., Cary, NC, USA) and R Statistical software (version 4.4.2; R Foundation for Statistical Computing, Vienna, Austria). The continuous variables are demonstrated as medians and interquartile ranges and were compared using the Wilcoxon Rank Sum Test. The categorical variables are presented as percentages and were compared using the chi-square test. The prognostic strengths of the ALBI score were compared by calculating the area under the curve (AUC), sensitivity and specificity. The optimal cut-off value for the ALBI score was estimated using the Youden criterion. The Kaplan–Meier curve with the log-rank test was performed to compare mortality in patients dichotomized according to their ALBI score, based on the optimal cut-off values. Moreover, a hazard ratio (HR) with 95% CI was calculated using the Cox regression model. We also performed additional sensitivity analysis by including an interaction term into the Cox regression to check whether the ALBI score remained predictive across subgroups, including the following: age, sex, ischemic etiology of HF, body-mass index, hypertension, type 2 diabetes, persistent atrial fibrillation and COPD. A 2-sided *p*-value of less than 0.05 was considered statistically significant.

## 3. Results

The study cohort consisted of 242 patients, with a median (IQR) age of 68.00 (range 66.0 to 74.6) years. 54.1% of the patients were in the NYHA III class and 45.9% were in the NYHA IV class. A total of 61.2% of the patients had documented persistent atrial fibrillation history, 55.8% were hypertensive and 38% were diabetic. The median left ventricular ejection fraction of the patients was 19.5 (15.0–23.0). On admission, 28.9% of patients required intropic drugs. The mean follow-up time was 352 ± 293 days. During the follow-up period, 47.1% patients died. A summary of the detailed clinical characteristics of the study population is provided in Table 1.

**Figure 1:**
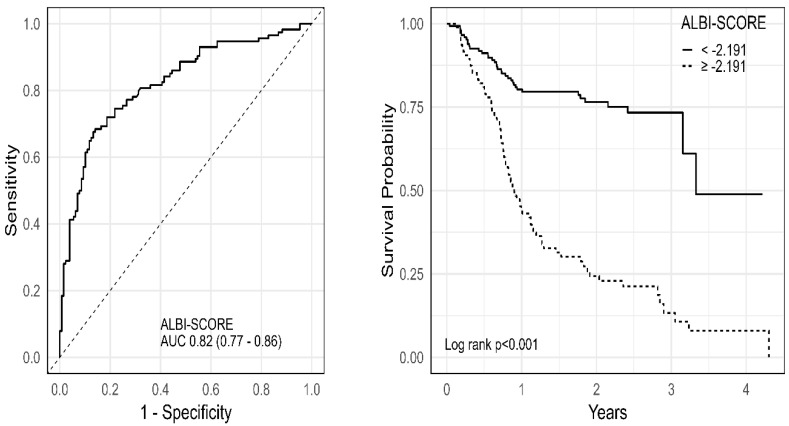
The ROC and Kaplan–Meier survival curves for ALBI score. Abbreviations: ALBI, albumin–bilirubin; AUC, area under the curve.

**Figure 2:**
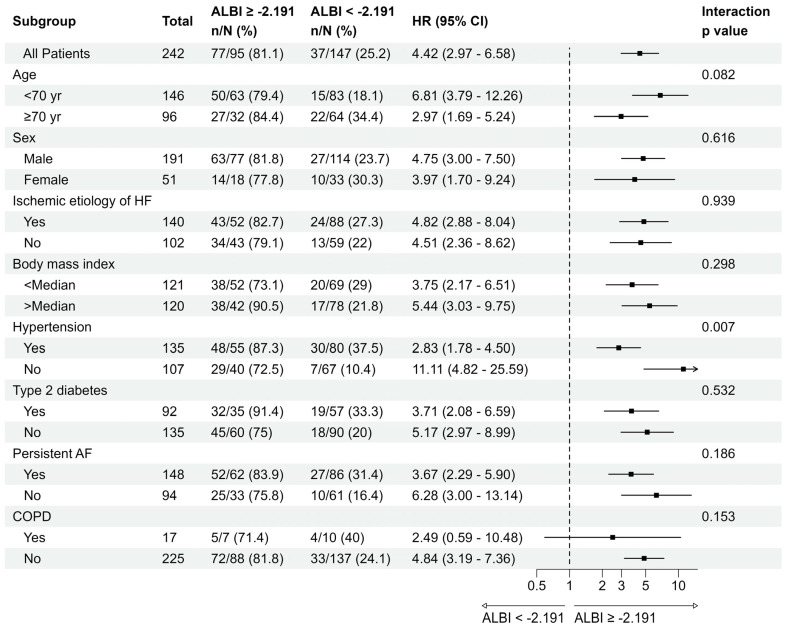
The subgroup analysis for the death outcome in comparison between lower (<−2.191) and higher (≥−2.191) ALBI scores. Abbreviations: see Table 1; CI, confidence interval; HR, hazard ratio.

The ALBI score generated good predictive power (AUC = 0.822, *p* < 0.01) and specificity (86%) as well as acceptable sensitivity (68%) to predict long-term mortality. The ROC curve for the ALBI scores for long-term mortality is demonstrated in Figure 1. The Kaplan–Meier survival curves to compare the survival of the patients dichotomized according to the ALBI score cut-off are also shown in Figure 1. Patients with higher ALBI scores (≥−2.191) had significantly worse long-term survival than patients with lower ALBI scores (<−2.191) [37 (25.2%) vs. 77 (81.1%); log rank *p* < 0.001]. The results obtained from the Receiver Operating Characteristic analysis for ALBI score are shown in Table 2. The median survival time (with 95% confidence interval) was 3.3 (3.2, −) and 0.9 (0.78, 1.1) years for patients with lower ALBI scores (<−2.191) and higher ALBI scores (≥−2.191), respectively. Table 3 presents the probability of 1, 2, 3 and 4 years of survival. The hazard ratio for patients with higher ALBI scores (≥−2.191) was 4.42 (95% CI: 2.97–6.58). Sensitivity analysis showed that the ALBI score remains predictive across subgroups (Figure 2).

## 4. Discussion

The present study demonstrated that the ALBI score had good predictive power and allowed the identification of elderly patients with end-stage HF at a high risk of death in long-term follow-up.

The study by Yamada et al. also showed that ALBI score was a useful marker for risk stratification and response to cardiac resynchronization therapy in HF populations [5].

In study [5], the analyzed population was of a similar age, but the patients had a higher LVEF and the percentage of persons in NYHA classes III and IV was relatively small (35%). During the 50-month follow-up, the percentage of deaths was lower (22.7%), which could result from the fact that the analyzed patients were in a lower stage of advancement of the disease. Kawata et al. also showed that the higher ALBI score on admission was an independent factor of higher in-hospital mortality in the patients hospitalized for acute HF [6]. However, the analyzed population differed fundamentally from ours as it included significantly older patients (median age 86 years) with a higher left ventricular ejection fraction [45% (35–61)]. Furthermore, the authors analyzed only in-hospital mortality (during hospitalization 14.1% of patients died); therefore, it was unknown whether the ALBI score would maintain prognostic value in the long-term follow-up [6]. In turn, Matsue et al. demonstrated that the ALBI score was an important marker of liver function and fluid overload in patients with acute HF, and its prognostic power at 1-year follow-up was higher than that of the well-known liver function scale Model for End-stage Liver Disease excluding INR (MELD-XI) [3]. In contrast to the study by Matsue et al. [3], the present study focused specifically on patients with advanced HF (NYHA III and IV, median left ventricular ejection fraction 19%) who had a more severe disease course, and focused on the long-term risk of all-cause mortality. Another study demonstrated that a higher ALBI score was also associated with HF severity, hepatic congestion and impairment due to right HF [9]. From the pathophysiological point of view, biomarkers of liver function may be useful indicators of worse prognosis in elderly patients with HF. ALBI score, which incorporates two simple and cheap clinical variables reflecting impaired synthesizing liver function (reduced albumin level) and impaired metabolic liver function (increased bilirubin level) secondary to HF exacerbation, may facilitate risk stratification in elderly patients with HF [3,4,5].

Our study has several limitations. Its single-center design limits generalizability. Furthermore, our analysis included a relatively small group of elderly patients with HF, hospitalized due to its decompensation. The exclusion of patients with severe liver disease or sepsis may result in the underestimation of the ALBI score’s full utility. Future studies should also determine the role of ALBI in the context of other prognostic indicators in elderly patients with HF. In our study, we also did not evaluate echocardiographic and hemodynamic phenotypes in our analyzed group of patients. The accurate assessment of such a profile can facilitate therapeutic decisions, and the most frequently observed cold–dry pattern in the elderly population (poor perfusion without pulmonary congestion) usually responds to volume and/or inotropic drugs [10]. Future studies should also investigate whether combining other volume overload factors, such as serum uric acid to estimated glomerular filtration ratio or urine albumin–creatinine ratio, with the ALBI score [11,12] can improve the prognostic value of the assessment of elderly patients with advanced HF hospitalized due to decompensation. Multi-center or randomized settings are necessary to confirm the prognostic value of ALBI score in analyzed groups of patients.

In summary, to the best of our knowledge, this is the first study which demonstrates that ALBI score has good prognostic power and allows for the successful prediction of mortality during long-term follow-up in elderly patients hospitalized due to the exacerbation of HF. This study may have clinical implications because it provides a simple and cheap indicator for risk stratification during hospital admission or outpatient follow-up.

## Figures and Tables

**Table 1:** The baseline characteristics of the study population.

	Overall n = 242	Survival Group n = 128	Non-Survival Group n = 114	*p* Value
Age, years	68.00 (66.00, 74.64)	67.76 (66.00, 73.42)	68.54 (66.00, 75.00)	0.382
Female, n (%)	51 (21.1)	27 (21.1)	24 (21.1)	0.994
Ischemic etiology of HF, n (%)	140 (57.9)	73 (57.0)	67 (58.8)	0.784
BMI, kg/m^2^	26.37 (23.73, 30.07)	26.42 (23.66, 30.18)	26.12 (24.20, 29.40)	0.871
Hypertension, n (%)	135 (55.8%)	57 (44.5%)	78 (68.4%)	<0.001
Type 2 diabetes, n (%)	92 (38.0%)	41 (32.0%)	51 (44.7%)	0.042
Persistent AF, n (%)	148 (61.2%)	69 (53.9%)	79 (69.3%)	0.014
COPD, n (%)	17 (7.0%)	8 (6.3%)	9 (7.9%)	0.617
Albumin, g/L	37.00 (34.00, 41.00)	40.00 (37.00, 42.00)	35.00 (33.00, 37.00)	<0.001
Total bilirubin, µmol/L	21.10 (14.60, 29.80)	18.10 (11.55, 25.55)	23.35 (18.48, 31.88)	<0.001
ALBI score	−2.35 (−2.63, −2.02)	−2.56 (−2.75, −2.34)	−2.06 (−2.33, −1.82)	<0.001
INR	1.41 (1.16, 2.00)	1.33 (1.08, 2.00)	1.45 (1.19, 2.00)	0.092
APTT, s	37.05 (32.38, 43.23)	36.90 (31.80, 42.65)	37.20 (32.70, 43.90)	0.494
Glucose, mmol/L	5.95 (5.30, 7.48)	5.80 (5.10, 7.20)	6.10 (5.30, 7.60)	0.087
Creatinine, µmol/L	121.50 (95.00, 149.00)	109.00 (87.00, 133.25)	135.00 (107.75, 177.75)	<0.001
Uric acid, µmol/L	420.00 (329.50, 536.00)	411.50 (313.75, 539.50)	432.00 (350.00, 531.00)	0.150
Cholesterol, mmol/L	3.51 (2.79, 4.33)	3.55 (2.81, 4.21)	3.50 (2.75, 4.46)	0.826
LDL, mmol/L	1.97 (1.41, 2.56)	1.98 (1.46, 2.58)	1.96 (1.41, 2.51)	0.754
Hemoglobin, mmol/L	9.60 (8.33, 12.45)	9.60 (8.40, 12.63)	9.80 (8.30, 12.25)	0.969
WBC, ×10^9^/L	7.00 (6.00, 9.00)	7.00 (6.00, 9.00)	7.00 (5.29, 9.00)	0.274
Platelets, ×10^9^/L	188.00 (147.25, 241.00)	198.50 (153.50, 250.00)	180.50 (140.50, 210.75)	0.022
NTproBNP, pg/mL	6352.50 (2887.25, 11403.25)	5904.00 (2403.25, 9710.00)	7646.00 (3625.50, 13495.00)	0.021
Sodium, mmol/L	137.00 (134.00, 140.00)	138.00 (135.00, 140.00)	136.00 (133.00, 139.00)	0.002
LVEDd, mm	68.50 (63.25, 75.00)	67.00 (64.00, 75.00)	69.00 (63.00, 75.00)	0.785
LA, mm	51.00 (47.00, 57.00)	50.00 (46.25, 54.75)	52.00 (48.00, 58.00)	0.023
LVEF, %	19.50 (15.00–23.00)	19.50 (14.00–25.00)	19.50 (15.00–21.00)	0.879
B-blockers, n (%)	225 (93.0%)	119 (93.0%)	106 (93.0%)	0.997
MRA, n (%)	225 (93.0%)	119 (93.0%)	106 (93.0%)	0.997
ACEI/ARB/ARNI, n (%)	189 (78.1%)	100 (78.1%)	89 (78.1%)	0.992
Flosins, n (%)	162 (66.9%)	86 (67.2%)	76 (66.7%)	0.931
Loop diuretics, n (%)	230 (95.0%)	120 (93.8%)	110 (96.5%)	0.327
Inotropic at admission, n (%)	70 (28.9%)	35 (27.3%)	35 (30.7%)	0.565
VKA, n (%)	74 (30.6%)	32 (25.0%)	42 (36.8%)	0.046
Digoxin, n (%)	66 (27.3%)	31 (24.2%)	35 (30.7%)	0.258
Statin, n (%)	129 (53.3%)	68 (53.1%)	61 (53.5%)	0.952
Acetylsalicylic acid, n (%)	63 (26.0%)	34 (26.6%)	29 (25.4%)	0.842
NOAC, n (%)	82 (33.9%)	56 (43.8%)	26 (22.8%)	<0.001

Data are presented as median (interquartile range) or mean (SD) unless otherwise indicated. Abbreviations: ACEI, angiotensin-converting enzyme inhibitor; AF, atrial fibrillation; ALBI, albumin–bilirubin; APTT, activated partial thromboplastin time; ARB, angiotensin receptor blockers; ARNI, angiotensin receptor/neprilysin inhibitor; BMI, body mass index; COPD, chronic obstructive pulmonary disease; HF, heart failure; INR, international normalized ratio; LA, left atrium; LDL, low-density lipoprotein; LVEDd, left ventricular end-diastolic dimension; LVEF, left ventricular ejection fraction; MRA, mineralocorticoid receptor antagonist; NOAC, non-vitamin K antagonist oral anticoagulants; NT-proBNP, N-terminal pro-B-type natriuretic peptide; NYHA, New York Heart Association; VKA, Vitamin K antagonists; WBC, white blood cells.

**Table 2:** A summary of the Receiver Operating Characteristic analysis for ALBI score.

	AUC [±95 CI]	Cut-off	Sens. [±95 CI]	Spec. [±95 CI]	PPV [±95 CI]	NPV [±95 CI]	Accuracy
ALBI score	0.822 [0.768–0.875]	−2.191	0.68 [0.59–0.77]	0.86 [0.79–0.91]	0.81 [0.72–0.87]	0.75 [0.67–0.84]	0.78 [0.74–0.83]

Abbreviations: AUC, area under the curve; CI, confidence interval; NPV, negative predictive value; PPV, positive predictive value.

**Table 3:** Probability of surviving beyond 1, 2, 3 and 4 years in patients with lower and higher ALBI scores. Results displayed are % with 95% CI.

Time (Years)	ALBI Score	
	<−2.191	≥−2.191
1 year	80.3 (74.1–87.0)	44.2 (35.3–55.4)
2 year	76.5 (69.7–84.0)	24.4 (16.7–35.6)
3 year	73.3 (65.7–81.9)	13.3 (6.73–26.4)
4 year	48.9 (27.5–87.0)	7.99 (2.97–21.5)

Abbreviations: ALBI, albumin–bilirubin ratio.

## Data Availability

The data presented in this study are available on request from the corresponding author. The data are not publicly available due to privacy restrictions related to the rules in our institution.
